# Misreporting of contraceptive hormone use in clinical research participants

**DOI:** 10.1016/j.contraception.2017.09.013

**Published:** 2018-04

**Authors:** Sharon L. Achilles, Felix G. Mhlanga, Petina Musara, Samuel M. Poloyac, Zvavahera M. Chirenje, Sharon L. Hillier

**Affiliations:** a Department of Obstetrics, Gynecology, and Reproductive Sciences and Center for Family Planning Research, University of Pittsburgh School of Medicine, Pittsburgh, PA, USA; b Magee-Womens Research Institute, Pittsburgh, PA, USA; c University of Zimbabwe–University of California at San Francisco Collaborative Research Unit (UZ–UCSF), Department of Obstetrics and Gynecology, Harare, Zimbabwe; d School of Pharmaceutical Sciences, University of Pittsburgh, Pittsburgh, PA, USA

**Keywords:** Self-report, Hormonal contraception, LARC, Misreporting, Oral contraceptive pills

## Abstract

**Objective:**

Researchers traditionally rely on participant self-report for contraceptive use. We hypothesized that self-reported contraceptive use by clinical research participants may disagree with objectively measured hormonal status.

**Study design:**

We enrolled women in Harare, Zimbabwe, aged 18–34, who by self-report had not used hormonal or intrauterine contraception for >30 days, or depot medroxyprogesterone acetate for >10 months, into a study designed to assess biologic changes with contraceptive initiation and use. Blood samples obtained at enrollment and each follow-up visit (*N*=1630 from 447 participants) were evaluated by mass spectrometry for exogenous hormones. We individually interviewed a subset of participants (*n*=20) with discrepant self-reported and measured serum hormones to better understand nondisclosure of contraceptive use.

**Results:**

Discrepant with self-reported nonuse of hormonal contraception, synthetic progestogens were detectable in 120/447 (27%, 95% confidence interval 23%–31%) enrolled women. Measured exogenous hormones consistent with use of contraceptive pills (*n*=102), injectables (*n*=20) and implants (*n*=3) were detected at enrollment, with 7 women likely using >1 contraceptive. In-depth interviews revealed that participants understood the requirement to be hormone free at enrollment (100%). Most (85%) cited partner noncooperation with condoms/withdrawal and/or pregnancy concerns as major reasons for nondisclosed contraceptive use. All interviewed women (100%) cited access to health care as a primary motivation for study participation. Of participants who accurately reported nonuse of hormonal contraception at enrollment, 41/327 (12.5%) had objective evidence of nonstudy progestin use at follow-up that disagreed with self-reported nonuse.

**Conclusions:**

Women joining contraceptive research studies may misrepresent their use of nonstudy contraceptive hormones at baseline and follow-up. Objective measures of hormone use are needed to ensure that study population exposures are accurately categorized.

**Implications statement:**

Among Zimbabwean women participating in a contraceptive research study, 27% had objective evidence of use of nonstudy contraceptives at enrollment that disagreed with self-report. Studies that rely on self-report to identify contraceptive hormone exposure could suffer from significant misclassification.

## 1 Introduction

Clinical research investigators traditionally rely on participant self-report for important variables including last menstrual period (LMP) and contraceptive use. Many published studies have relied on these self-reported variables to critically classify participants into analysis cohorts on which outcomes are determined [1], [2], [3], [4], [5], [6], [7], [8], [9], [10], [11]. Objective biomarkers of exposure have rarely been evaluated. Recently, several authors have described significant discrepancies between self-report and objective biomarker exposure data for sexual activity [12], [13], history of *Chlamydia trachomatis* infection [14], tobacco use [15] and contraceptive use [16], [17], [18]. Misreported contraceptive use could bias results in studies examining the effects of specific contraceptives, for instance, studies of contraceptive injectables and human immunodeficiency virus (HIV) acquisition risk [19]. Research to validate self-reported contraceptive use is limited [20].

Studies assessing use of hormonal contraception and HIV acquisition risk show mixed outcome data [19]. In order to untangle possible biological links between hormonal status and risk of acquiring sexually transmitted infections (STIs) and HIV, there is a need to understand if self-reported variables are adequate for cohort assignment. We hypothesized that self-reported contraceptive use by clinical research participants may disagree with objectively measured hormonal status. In order to assess accuracy of self-reported LMP and contraceptive use, we compared laboratory evaluation of serum progestogens and estrogens to participant self-report. We also explored reasons for misreporting in a subset of participants with discrepant self-reported and measured serum hormone data.

## 2 Materials and methods

### 2.1 Study population and sample collection

We performed a parallel cohort study (ClinicalTrials.gov number: NCT02038335) of women initiating contraception with injectable [depot medroxyprogesterone acetate (DMPA), norethisterone enanthate (Net-En), medroxyprogesterone acetate and ethinyl estradiol (MPA/EE)], implant [levonorgestrel subdermal implant (LNG-I) or etonogestrel subdermal implant (ENG-I)] or intrauterine [copper T380A intrauterine device (Cu-IUD)] contraception. The primary objective was to assess the impact of initiation and continued use of contraceptives on HIV target cells in the lower genital tract at 1, 3 and 6 months of use. The study was designed to assess changes compared to baseline with each woman serving as her own control; therefore, being free of exogenous steroid hormones at baseline and in a uniform phase of menses was central to the study design. Given the critical importance of the baseline values, laboratory confirmation by ultra-high-performance liquid chromatography tandem mass spectrometry (UPLC/MS/MS) was performed to evaluate serum progesterone (P4), levonorgestrel (LNG), etonogestrel (ENG), norethindrone (NET) and medroxyprogesterone acetate (MPA) concentrations, which covered the full spectrum of regionally available contraceptive progestins at the time this study was conducted. Baseline sampling was performed at the enrollment visit when all enrolled women were free of hormonal or intrauterine contraceptive use for the preceding 30 days and free of DMPA use for the preceding 10 months by self-report. The University of Pittsburgh Institutional Review Board and The Medical Research Council of Zimbabwe approved this study. All participants were enrolled at Spilhaus Family Planning Centre in Harare, Zimbabwe, and signed informed consent before study participation.

Enrollment consisted of 451 women, age 18–34 years, seeking contraception in Harare, Zimbabwe. Eligible women were healthy, HIV negative and nonpregnant and had regular menstrual cycles. Women were excluded if within 30 days of enrollment they (1) used any hormonal or intrauterine contraceptive; (2) underwent any genital tract procedure (including biopsy); (3) were diagnosed with any urogenital tract infection; or (4) used any oral or vaginal antibiotics, oral or vaginal steroids, or any vaginal product or device except tampons and condoms (such as spermicide, microbicide, douche, sex toys and diaphragms). Women were also excluded if by self-report they used DMPA within 10 months of enrollment, were pregnant or breastfeeding within 60 days of enrollment, or had a new sexual partner within 90 days of enrollment. Exclusion criteria included having a contraindication, allergy or intolerance to use of the contraceptive desired by the participant and having a prior hysterectomy or malignancy of the cervix or uterus.

Screening included urine pregnancy testing; two rapid HIV screening tests to rule out HIV infection; and collection of genital tract swabs for detection of *Neisseria gonorrhoeae*, *Chlamydia trachomatis* (Cepheid, Sunnyvale, CA, USA) and *Trichomonas vaginalis* (OSOM, Sekisui Diagnostics, Lexington, MA, USA).

Eligible participants presented for enrollment on a day when no vaginal bleeding was present and when they were in the follicular phase of menses (day 1–14) by self-reported LMP. Participants were asked to refrain from any vaginal or anal intercourse for 48 h prior to sample collection at enrollment and all follow-up visits. Participants selected their contraceptive group from among the six options (DMPA, Net-En, MPA/EE, LNG-I, ENG-I and Cu-IUD), and the selected contraceptive was administered by a study clinician at the enrollment visit immediately following collection of all study samples. IUDs and implants were inserted per standard clinical practice. All laboratory personnel were masked to clinical status of participants including contraceptive group.

### 2.2 Laboratory methods

Collection of blood and genital tract samples occurred at enrollment and at follow-up visits on days 30, 90 and 180. Blood was collected in 4-mL tubes (Becton Dickinson, Franklin Lakes, NJ, USA) and transported to the UZ–UCSF Central Laboratory on ice within 90 min. Specimen identifier details were entered into Laboratory Information System (DISA Laboratory Information System, Laboratory System Technologies, version 16.03), and unique identifiers were generated and assigned for subsequent processing. Blood samples were centrifuged at 1100*g* for 10 min at 20°C. Serum was harvested and aliquoted into 2-mL cryo vials (SARSTEDT Aktiengesellschaft & Co., Nümbrecht, Germany) and immediately transferred to −80°C for storage pending shipment for centralized hormonal testing at the University of Pittsburgh. Laboratory Data Management System (Frontier Science Research & Technology foundation, Buffalo, NY, USA) mapped freezer storage positions of all aliquots, and shipping batches were created monthly for specimen shipment to Magee-Womens Research Institute on dry ice following International Air Transport Association regulations.

Blood samples (*N*=1630 from 447 enrolled participants) were evaluated by mass spectrometry for contraceptive hormones at enrollment and each follow-up visit. UPLC/MS/MS was used for quantification of estrogens [21] and progestins [22] as previously described, respectively, with modifications. Generally, UPLC/MS/MS employs liquid–liquid extraction, derivatization (for estrogens) and detection in the positive mode with a Thermo Fisher TSQ Quantum Ultra mass spectrometer with the Waters UPLC Acquity solvent delivery system. Serum (0.5 mL), spiked with two internal standards (d5-17 beta-estradiol and d3-testosterone), was extracted with n-butyl chloride. After centrifugation and evaporation, the residue was reconstituted in 50 μL of methanol:water (50:50) and split for analysis on estrogen and progestogen panels.

For the progestin panel, half (25 μL) of the residue was transferred to autosampler glass vials for injection. Progestins were eluted from a Waters Acquity UPLC BEH C18, 1.7-μm, 2.1 × 150-mm reversed-phase column, with a methanol:water (0.1% formic acid and 2 mM ammonium acetate) gradient. The lower limit of quantitation for all progestins was 25 pg/mL. The mass to charge transitions used for analysis of each progestin were as follows: ENG (325➔257), LNG (313➔245), MPA (387➔327), NET (299➔231), P4 (315➔109) and d3 testosterone (292➔97). Participants were considered to be not in the follicular phase of the menstrual cycle with P4 ≥1000 pg/mL.

For the estrogen panel, half (25 μL) of the residue was evaporated and derivatized in 0.1-mL buffered dansyl chloride solution (pH 10.5), and transferred to autosampler glass vials for injection. Estradiol was eluted from a Waters Acquity UPLC BEH C18, 1.7-μm, 2.1 × 150-mm reversed-phase column, with an acetonitrile:water (0.1% formic acid) gradient. The lower limit of quantitation for all estrogens was 10 pg/mL. Mass spectrometry detection was conducted via heated electrospray ionization with a spray voltage of 4000 V, vaporizer temperature 355°C, sheath gas 20 units, a capillary temperature 350°C and a collision energy of 35 V for each metabolite. The mass to charge transitions used for analysis of each metabolite were as follows: estradiol (506➔171), ethinyl estradiol (530➔171) and 2,4,16,16,17-d5-17 beta-estradiol (511➔171).

All samples from participants found to have exogenous synthetic progestin blood levels at enrollment contradictory to self-reported nonuse were retested to confirm biological results and to rule out contamination during sample processing. All retesting confirmed original results, and the participants were disqualified from continued study participation.

### 2.3 Interviews with participants found to have discrepant objective and self-reported results

A subset of 20 consecutive participants who came to the study site for study termination/disqualification between March 3 and 6, 2015, due to discrepant objective and self-reported hormone results were invited for in-depth one-on-one interviews in their native language to better understand their nondisclosure of contraceptive use. These interviews were all conducted by a single, independent, local investigator who was not part of the study team (P.M.). Participants were asked four structured open-ended questions exploring their motivations to join this study and their understanding of the exclusion criteria, specifically regarding use of hormonal contraceptives within 30 days of enrollment. The interviewer then told these participants their specific blood test results and recorded their reactions. Women were asked their reasons for continuing to take contraceptives and to speculate about why some women may have used contraceptives and not disclosed use to study staff. For analysis, common themes were identified, tabulated and reported.

### 2.4 Statistical analysis

Descriptive statistics including frequencies, medians and means with standard deviation were used to characterize demographic and behavioral characteristics. Student's *t*, Mann–Whitney *U* or Fisher's Exact Tests were used as appropriate to compare evaluable and disqualified participants. Nonstudy contraceptive use was compared between study arms using Fisher's Exact Test, and exact binomial 95% confidence interval (CI) was calculated.

## 3 Results

### 3.1 Demographic characteristics

**Fig. 1:**
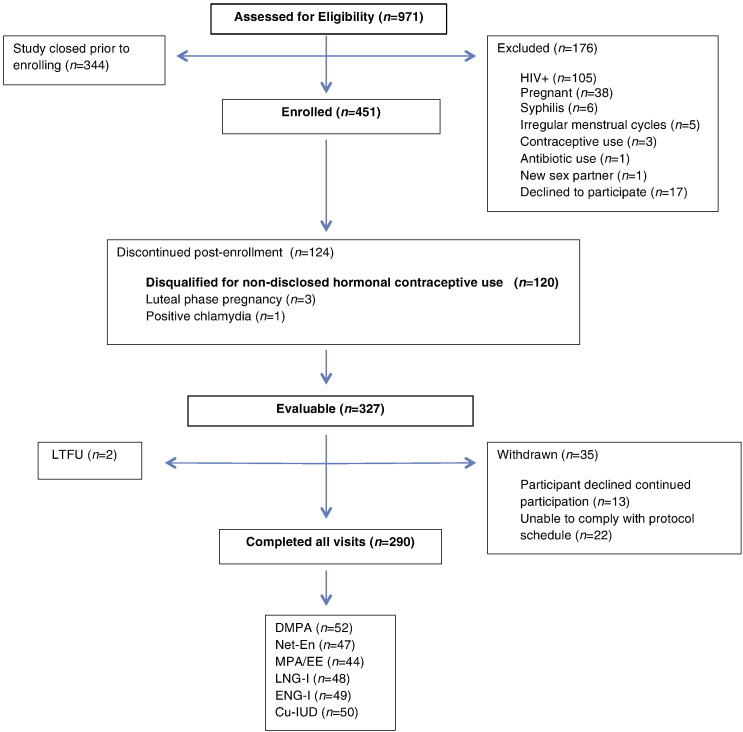
Study flowchart. A diagram of participant flow from eligibility assessment to final categorization.

Between February 2014 and December 2015, 971 participants were assessed for study eligibility and 451 were enrolled. Of the 451 enrolled participants, 120 (27%) were disqualified after enrollment for nondisclosed hormonal contraceptive use, 4 (<1%) were otherwise found ineligible after enrollment, and 327 (73%) were evaluable. A flow diagram of all screened and enrolled participants is shown in Fig. 1.

Overall, enrolled participants who accurately reported (evaluable) compared to misreported (disqualified) contraceptive use at enrollment did not differ on any demographic or sexual behavioral feature (Table 1) except that a greater proportion of women misreporting hormonal status at enrollment reported oral contraceptive pill use at screening compared to evaluable enrollees (79.2% vs. 66.4%, p=.01).

**Table 1:** Demographic characteristics

	Enrolled participants		
	Evaluable (*n*=327)	Disqualified (*n*=120)	p value
Age, years	27.0±4.1	26.2±3.9	.06^a^
Gravidity (median, IQR)	2 (1–3)	2 (2–3)	.34^b^
Parity (median, IQR)	2 (1–3)	2 (1–3)	.39^b^
Body mass index (kg/m^2^)	25.5±4.8	25.7±4.7	.74^a^
Ethnicity			.83^c^
Shona	305 (93.3%)	111 (92.5%)	
Ndebele	9 (2.8%)	3 (2.5%)	
Malawian	12 (3.7%)	6 (5.0%)	
Zambian	1 (0.3%)	0	
Marital status			.35^c^
Single (never married)	11 (3.4%)	1 (0.8%)	
Married	276 (84.4%)	110 (91.7%)	
Divorced	24 (7.3%)	5 (4.2%)	
Separated	13 (4.0%)	4 (3.3%)	
Widowed	3 (0.9%)	0	
Partner status			.23^c^
Lives with partner	272 (83.2%)	108 (90.0%)	
Does not live with partner	47 (14.4%)	11 (9.2%)	
Not applicable/none	8 (2.4%)	1 (0.8%)	
Religious identification			.90^c^
Christian	307 (93.9%)	112 (93.3%)	
Muslim	6 (1.8%)	3 (2.5%)	
African traditional religion	2 (0.6%)	0	
None	12 (3.7%)	5 (4.2%)	
Education			.31^c^
None	1 (0.3%)	0	
Primary	41 (12.5%)	19 (15.8%)	
Secondary	273 (83.5%)	100 (83.3%)	
Tertiary	12 (3.7%)	1 (0.8%)	
Frequency of condom use in last 10 sexual encounters			.44^c^
0	234 (71.6%)	85 (70.8%)	
1–9	55 (16.8%)	25 (20.8%)	
10	38 (11.6%)	10 (8.3%)	
Typical frequency of intercourse (per month)	13.5±7.2	14.1±5.5	.46^a^
STIs at screening			
*Chlamydia trachomatis*	22 (6.7%)	7 (5.8%)	.83
*Neisseria gonorrhoeae*	10 (3.1%)	2 (1.7%)	.53
*Trichomonas vaginalis*	24 (7.3%)	10 (8.3%)	.69
Contraceptive pill use reported at screening^d^	217 (66.4%)	95 (79.2%)	.01

Data presented as mean ± standard deviation or *n* (%).

a p value from Student's *t* test.

b p value from Mann–Whitney *U* test.

c p value from Fisher's Exact Test.

d All participants reporting contraceptive pill use at screening agreed to discontinue use during the screening window in order to be hormonal and intrauterine contraceptive free for>30 days at enrollment in accordance with the study protocol.

### 3.2 Misreporting of contraceptive use

Overall, 120/447 (27%, 95% CI 23%–31%) of participants who reported no contraceptive use at baseline had objective evidence of hormonal contraceptive use at enrollment, and 161/447 (36%) had objective evidence of nonstudy hormonal contraceptive use at any visit. Table 2 displays the frequency and median serum progestin concentration of misreported nonstudy hormonal contraceptives at baseline (enrollment) and each follow-up visit for all enrolled participants inclusive of those ultimately disqualified for nondisclosed hormonal contraceptive use (*n*=447).

**Table 2:** Detection of nonstudy contraceptive use in sera among enrolled participants

Visit	LNG		ENG		NET		MPA	
	*n* (%)	[LNG]^a^	*n* (%)	[ENG]^a^	*n* (%)	[NET]^a^	*n* (%)	[MPA]^a^
Baseline	102/447 (23%)	2634 (27–23,839)	2/447 (<.5%)	1156 (62–2249)	2/447 (<.5%)	36 (29–43)	20/447 (4.5%)	189 (26–1877)
30 days	39/431 (9%)	2714 (118–22,272)	4/431 (<1%)	227 (60–1177)	1/431 (<.5%)	38	9/431 (2%)	131 (31–1626)
90 days	36/403 (9%)	2280 (45–22,836)	3/403 (<1%)	783 (253–1414)	0	–	3/403 (<1%)	85 (60–88)
180 days	28/349 (8%)	1039 (79–23,160)	4/349 (1%)	508 (288–1832)	1/349 (<.5%)	712	2/349 (<1%)	834 (38–1630)

a Median nonstudy progestin serum concentration in pg/mL (range).

### 3.3 Qualitative interviews with misreporting participants

Twenty consecutive participants reporting for their study termination visit were approached, and all agreed to participate in qualitative interviews that lasted an average of 27 min each (range 17–38 min). Table 3 lists the four structured questions that were asked and the tabulated responses. All interviewed participants reported a clear understanding of the study requirement to be contraceptive-free at enrollment. During the interviews, 10/20 (50%) expressed a strong desire to avoid pregnancy, and 16/20 (80%) noted that their partner disagreed with or refused to use condoms for some or all the time prior to enrollment. Thus, women may have persisted with oral contraceptive use at baseline to avoid pregnancy. All interviewed participants discussed access to free health services as a primary motivator for seeking study participation, and during the interviews, 3/20 (15%) admitted misreporting contraceptive use at baseline in order to qualify for study participation by ensuring that they were not pregnant at enrollment. An additional 11/20 (55%) stated that it “could be possible” that hormones were found in their blood samples. The LNG levels at enrollment for 6 participants who strongly denied hormonal contraceptive use for the ≥30 days between screening and enrollment were 3486 pg/mL, 6792 pg/mL, 5205 pg/mL, 10,600 pg/mL, 2105 pg/mL and 6379 pg/mL.

**Table 3:** Qualitative interviews with participants with discrepant self-reported and measured contraceptive hormone use

What were your reasons for joining this study?	
Access to free health care services	20/20 (100%)
HIV/STI screening	15/20 (75%)
Access to family planning services	15/20 (75%)
Cervical cancer screening	15/20 (75%)
Altruistic reasons	3/20 (15%)
Pregnancy testing	1/20 (5%)
Encouraged by a friend	1/20 (5%)
Women participating in this study were asked to stop all hormonal contraceptive use between screening and enrollment. How did you understand this instruction?	
Instructions to not use any hormonal contraception were clear	20/20 (100%)
Partner was also informed by study staff and agreed	6/20 (30%)

The results of your blood test showed the presence of contraceptive hormones in your system at enrollment; we'd like your help understanding these results. What do these results mean to you and what are all of the reasons that you were able/not able to stop hormonal contraception before enrollment in this study?	
Partner disagreement or refusal to use condoms/withdrawal	16/20 (80%)
Hormone did not clear system by 30 days	11/20 (55%)
Received and used free condoms	11/20 (55%)
Strong desire to avoid pregnancy	10/20 (50%)
No challenges to stopping hormonal contraception	10/20 (50%)
Partner was out of town/did not have sex during screening	4/20 (20%)
Admitted taking some contraceptive pills during screening	3/20 (15%)
Did not trust partner/fear of partner	2/20 (10%)
Did not want to disappoint study staff	2/20 (10%)
30 days is too long	1/20 (5%)
Did not disclose participation to partner	1/20 (5%)
Suspected laboratory error	1/20 (5%)
What could the study staff have done differently to get more honest or accurate responses on contraceptive use?	
No recommendations/changes needed	9/20 (45%)
Be open to problem-solving so participants can still enroll in study	7/20 (35%)
Emphasize and discuss common challenges to stopping hormonal contraception	4/20 (20%)
Help getting buy-in from partners	4/20 (20%)
More time for conversations with participants/avoid overscheduling	4/20 (20%)
Inform participants about the sensitivity of the testing (even 1 pill can be detected)	3/20 (15%)

### 3.4 Use of nonstudy contraceptives over time among women free of exogenous hormones at baseline

**Fig. 2:**
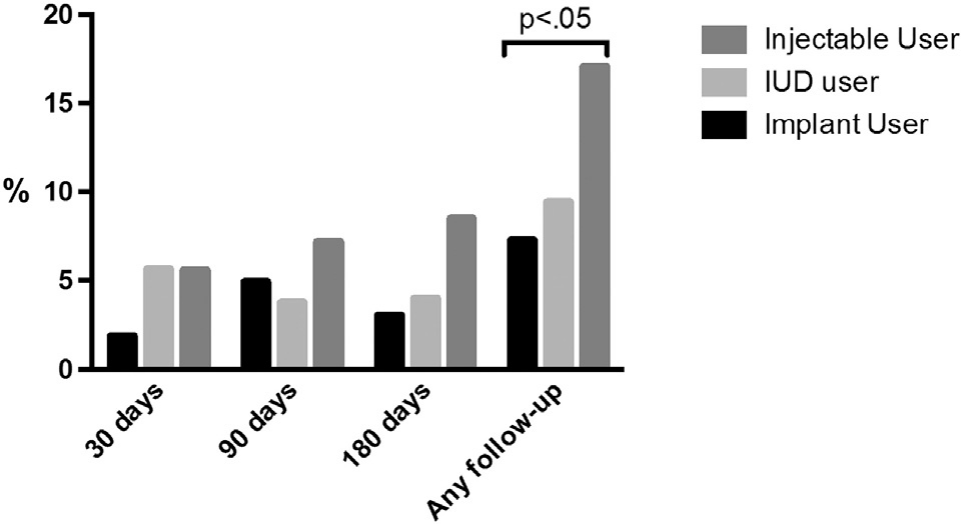
Proportion of women free of exogenous hormones at baseline (*N*=327) who had nonstudy hormones detected during follow-up. At enrollment, participant-selected study contraception was administered from the available options including injectables (DMPA, Net-En or MPA/EE), implants (LNG-I or ENG-I) or IUD (copper T380A). Participants were followed up at 30, 90 and 180 days after enrollment, and all reported no additional hormonal contraceptive use.

An analysis was done to evaluate whether use of nonstudy hormones occurred among the 327 enrolled participants who had accurately reported no contraceptive use at enrollment despite concern for pregnancy as was clearly expressed by participants in the qualitative interviews. Overall, 41/327 (13%) of these women had objective evidence of nonstudy contraceptive use at one or more follow-up visits. As shown in Fig. 2, women choosing injectable contraceptives were more likely to use nonstudy contraceptives compared to long-active reversible contraceptive (contraceptive implant or copper IUD) users (17% vs. 8%, p<.05, respectively).

### 3.5 Accuracy of LMP in predicting menstrual phase

We predicted that all participants were in the follicular-ovulatory phase of menses at enrollment based on being within the first 14 days of their menstrual cycles by self-reported LMP and regular 21–35-day menstrual cycles. We found that 54/447 (12%) were nonfollicular at baseline with mean P4=5577 pg/mL and median P4=4924 pg/mL. The remaining 393 participants had mean and median P4 values of 59 pg/mL and 35 pg/mL, respectively.

## 4 Discussion

In our study, we found frequent discrepancies between self-reported and measured serum contraceptive hormone use. In our cohort, interviewed participants reported a high desire to participate in research to gain access to free, high-quality health services, including HIV/STI testing and treatment, cervical cancer screening, pregnancy testing and access to family planning services, as well as clear understanding of the study requirements, suggesting that misreporting may have been purposeful.

Misreported contraceptive use could bias results in studies examining the effects of specific contraceptive use, for instance, studies of contraceptive injectables and HIV acquisition risk; however, there is limited research to validate self-reported use [20]. The few available published studies evaluating self-report for contraceptive use [16], [17], [18] suggest large discrepancies between self-report and objective measures, including up to 14% discrepant use in phase 3 contraceptive efficacy trials, where contraceptive pills use is thought to represent “perfect use” [17]. Further complicating analysis of self-reported contraceptive exposure is the common practice of women frequently starting, stopping and switching contraception, particularly user-controlled methods such as pills, patches and rings. We sought to understand the frequency of inaccurate self-reported contraceptive use by comparing biological testing to self-report within the context of a study evaluating genital tract immune cell response to contraceptive progestin initiation and use.

Inconsistencies between self-report and objective testing of contraceptive use generally did not appear to be due to slow metabolism and “hormonal tails” after a prescribed period of nonuse; rather, the vast majority of misreport was associated with participants having serum hormone levels indicative of active, steady-state use. Most nonstudy contraceptive use at enrollment was LNG based and consistent with locally available contraceptives and the observed serum values; this likely largely represents oral contraceptive pill use (102/120=85%). There are 2 varieties of regionally available contraceptive pills: one containing 0.075 mg norgestrel USP and one containing 0.30 mg norgestrel USP and 0.03 mg ethinyl estradiol. The minimum steady-state serum concentration with typical use of similarly dosed LNG-based oral contraceptive pills is ~2000 pg/mL [23], [24], [25], [26], [27]. In comparison, the steady-state serum LNG concentration in women using LNG-I is ~500 pg/mL. The median measured levels of nonreported LNG use were therefore consistent with active, ongoing use of oral contraceptive pills (Table 2). At enrollment, 20/120 (17%) participants with discordant self-reported and measured progestins had MPA measured, consistent with ongoing, active use of DMPA, which is also readily available locally. Very few participants, 4/120 (3%) had ENG or NET detected at enrollment, which is consistent with contraceptives such as Net-En, ENG-I and ENG vaginal rings not being locally available. Self-reported LMP used to predict menstrual phase, which requires regular menses and diligent tracking, was accurate 88% of the time in this study, suggesting that accuracy of self-report may improve when participants do not perceive the answer to jeopardize their study eligibility.

We are perplexed by the participants who accurately reported no hormonal contraceptive use at baseline (when they may have had increased risk of unintended pregnancy) yet initiated and actively used nonstudy short-acting contraceptives after study enrollment and initiation of a highly effective long-acting contraceptive method. The additional use of a less-effective, short-acting contraceptive would not add significantly to the contraceptive efficacy afforded by the study-provided long-acting methods. We therefore would have predicted that participants in the Cu-IUD or contraceptive implant groups may have opted to self-treat heavy or irregular menstrual bleeding that more frequently occurs with these methods compared to injectable contraceptives. Interestingly, a larger proportion of participants who self-selected injectable contraceptive use for the study used additional nonstudy hormonal contraceptives compared to IUD or implant users (17%, 9% and 7%, respectively; p<.05). Given that participants self-selected their study contraceptive group, this may suggest inherent differences among women who prefer injectables.

This study is likely generalizable to clinical study participants in Sub-Saharan Africa who are similarly being asked to self-report contraceptive use in a study reliant on contraceptive exposure, in other words, when there is a “right” and “wrong” answer for continued study participation. Many studies ask participants to self-report contraceptive use, and their answers would be recorded and otherwise nonlimiting for continued participation. Often, these self-reported contraceptive exposure data are then used in secondary analyses. This study may not be generalizable to such populations. Additionally, accuracy of self-report may be culturally influenced, and therefore, this study may not be generalizable to culturally disparate populations.

Overall, only 64% of all samples were associated with accurate reporting of contraceptive use, and women opting for use of injectable contraceptives were less likely to accurately report their contraceptive use. Verification of self-reported contraceptive use may reduce bias and may be critical for studies in which outcome data are reliant on contraceptive exposures. Biologic verification of self-reported exposures, including contraceptive use, adds considerable cost to the conduct of research studies and therefore may not be warranted for all studies, particularly those in which the outcome of interest does not depend on the self-reported exposure. Researchers conducting secondary analyses and systematic reviews that include studies with self-reported exposures must be alert to the potential introduction of bias and confounding.

## Funding

This work was supported by the Bill & Melinda Gates Foundation, Seattle, WA (grant number OPP1055833).

## Conflicts of interest

The authors report no conflicts of interest.
